# Enhancement of Film Cooling Effectiveness in a Supersonic Nozzle

**DOI:** 10.3390/e25030481

**Published:** 2023-03-10

**Authors:** Nithin Somasekharan, A. R. Srikrishnan, Harihara Sudhan Kumar, Krishna Prasad Ganesh, Akram Mohammad, Ratna Kishore Velamati

**Affiliations:** 1 Department of Aerospace Engineering, Amrita School of Engineering Coimbatore, Amrita Vishwa Vidyapeetham, Tamil Nadu 641112, India; 2 Department of Aerospace Engineering, King Abdulaziz University, Jeddah 21589, Saudi Arabia; 3 Department of Mechanical Engineering, Amrita School of Engineering Coimbatore, Amrita Vishwa Vidyapeetham, Tamil Nadu 641112, India

**Keywords:** rocket nozzle cooling, mixing layer studies, diffuser-type coolant injector, film cooling

## Abstract

Film cooling as applied to rocket nozzles is analyzed numerically with emphasis on the assessment of the effect of the mixing of coolant with the hot stream. Cooling performance, as characterized by cooling effectiveness, is studied for three different coolants in the three-dimensional, turbulent flow field of a supersonic convergent-divergent nozzle operating with a hot stream temperature of 2500 K over a range of blowing ratios. The coolant stream is injected tangentially into the mainstream using a diffuser-type injector. Parameters influencing the effectiveness, such as coolant injector configuration and mixing layer, are analyzed. Thermal and species mixing between the coolant and the mainstream are investigated with regard to their impact on cooling effectiveness. The results obtained provide insight into the film cooling performance of the gases and the heat transfer characteristics associated with these three gases. An injector taper angle of 30° results in the most effective cooling among the configurations considered (0°, 15°, 30° and 45°). Mixing of the coolant with the hot stream is examined based on the distributions of velocity, temperature and species. The higher values of cooling effectiveness for Helium are attributed to its thermophysical properties and the reduced rate of mixing with the hot stream. The results further indicate that through optimization of the blowing ratio and the coolant injector configuration, the film cooling effectiveness can be substantially improved.

## 1. Introduction

The efficiency and reliability of rocket engines have increased considerably over the past few decades. The continuously increasing demand for higher levels of propulsive thrust entails the need for operating the thrust chamber at significantly high levels of temperature, often exceeding 3000 K [1]. This necessitates that the thermal load on the internal walls of the rocket nozzle needs to be managed effectively in order to ensure the structural integrity of the system.

Film cooling is used, in combination with other methods of thermal load management, to provide a protective layer of coolant fluid to the surfaces that are continuously exposed to streams of hot combustion gases. The coolant is injected adjacent to the inner surface of the thrust chamber wall through small holes in such a way that it forms a thin film of relatively cool fluid, which acts as a layer of thermal insulation, isolating the surface from the mainstream of hot gases [2]. Near the location of injection, the secondary coolant forms a protective film of thermal insulation adjacent to the surface of the wall. Downstream of this location, the injected coolant continues to serve as a heat sink that brings down the local temperature level within the boundary layer [3]. Several existing rocket engines and gas turbines employ this method of cooling.

Many studies have addressed issues related to fluid dynamics and heat transfer in the basic mechanism of film cooling. Based on consideration of the development of the velocity field downstream of the coolant injection, Seban and Back [4] divide the film cooling flow field into three regions: (i) a core region which can be considered to be mostly inviscid, (ii) a region near the wall characterized by wall-jet behavior, and (iii) a region within the boundary layer. The growing boundary layer of the slot-jet merges with the outer layer of the inviscid core. An experimental study by Goldstein et al. [5] using a backward-facing slot for coolant injection showed that injection of air at supersonic velocity significantly increased the film cooling effectiveness in the near field of the injector, in comparison with subsonic injection. In addition, the experiments using Helium as a coolant showed that the higher value of specific heat of the coolant also enhanced the cooling effectiveness.

Gartshore et al. [6] carried out experiments to investigate the effect of the injection hole geometry on film cooling. Coolant injection from a circular hole improved cooling effectiveness in comparison to that with a square hole, as the enhanced rate of mixing with the hot stream impaired the cooling in the latter case. Optimizing the shape of the injectant hole was shown to have a significant impact on the cooling effectiveness in a recently reported experimental study with flat plate geometry [7]. Studies by Seban et al. [8] have shown a decrease in film cooling effectiveness due to the angling of the injectors primarily due to accelerated mixing with the mainstream. There was about a 50% drop in film cooling effectiveness for a normal injection as compared to a tangential injection due to the rapid mixing of the hot stream and the cold stream. The impact of the angularity of coolant injection on the effectiveness of a gaseous coolant was studied in detail by Shine et al. [9,10]. Increased mixing of the coolant with the mainstream was found to impede wall cooling for injection angles around 45°. The effects of wall conduction and blowing ratio $\left(BR=\frac{\rho_{c}v_{c}}{\rho_{h}v_{h}}\right)$ were also analyzed in the study. Tangential injections have several advantages, particularly in the context of supersonic nozzle cooling [11]. Tangentially injected jets produce minimum disturbance within the nozzle flow field, and the associated shock losses are relatively low. Other benefits include skin friction reduction and energization of the boundary layer. In a recent study, consisting of experimental and numerical characterization of film cooling, Laroche et al. [12] examined the impact of a high blowing ratio on cooling effectiveness—the thermal protection was found to be lower in the near field of the holes at higher values of the blowing ratio.

Most of the experimental heat transfer studies have utilized a single injection site and flat plates. A flat plate configuration implies that the study is conducted in the absence of a free stream pressure gradient. In internal flows, such as those that occur inside a rocket nozzle, the wall jet is expected to have a more prominent effect on the flow field [11,13]. Nozzle cooling systems are characterized by low injection angles, usually less than about 30° with respect to the surface.

The process of mixing between the injected coolant film and the mainstream fluid can have a critical impact on the extent of cooling and the cooling effectiveness [14]. The mixing of the film with the main flow occurs first through a mixing layer [15]. Subsequently, the mixing layer merges with the boundary layer, and an inviscid core initially fills the region between the wall boundary layer and the mixing layer. It progressively becomes thinner as the mixing layer spreads. Based on a comprehensive experimental study of film cooling in supersonic flow, Aupoix et al. [16] observed that the growth of the mixing layer can be controlled through the density ratio and velocity in order to improve the cooling effectiveness.

The transport properties of the coolant gas can have a significant impact on the cooling effectiveness. Mizoguchi et al. [17] studied the influence of the coolant gas properties on the film cooling effectiveness. The numerical simulations showed Helium to be a very effective coolant for managing the thermal load that results from aerodynamic heating in high enthalpy flows.

The numerical analysis reported by Kiran et al. [18] focuses on the impact of compressibility on mixing as applied to the wall jet along a flat plate. The study showed that the growth rate of the shear layer between the hot and the cold streams is significantly influenced by compressibility. A recent numerical study by Xiang and Sun [19] incorporating the effect of fuel injection and combustion on film cooling showed that vortex structures formed in the regions close to the injectors enhance the mixing of the coolant with the mainstream and thereby adversely impact the cooling effectiveness.

The impact of the interaction between the wall jet and the mainstream on cooling performance under different conditions has been analyzed in various studies ([20,21,22,23]). All these studies pertain to flat/cylindrical surface configurations. In a recent experimental study, which notably incorporates many of the real working conditions in a rocket nozzle, Ludescher and Olivier [24] showed that the increased height of the injection slot favored film cooling efficiency. The study also highlighted the influence of fluid properties, such as specific heat and molecular mass, on cooling performance. Verma et al. [13] numerically analyzed the impact of the location of coolant injection and the injection Mach number on the flow separation inside a film-cooled dual-bell nozzle—the study showed that both these parameters actively control the point of separation. The interaction of a liquid-phase film coolant and the core flow was studied numerically for a semi-cryogenic configuration by Strokach et al. [25]. In a recent numerical study, Guealalia et al. [26] explored the benefits of a novel approach to film injection, namely, a ‘lidded hole’ shape. The method was found to have the potential to improve cooling effectiveness significantly. Direct numerical simulation of film cooling as applied to a flat plate with a vertical slot has been reported by Peter and Klooker [27]. Film cooling in a backward-facing step configuration in which the coolant is injected along a flat wall was investigated numerically by Sargunaraj et al. [28]. The mainstream flow was at Mach 2.44, while the coolant injection Mach number varied from 1.2 to 2.2. The study highlights the impact of shock-boundary layer interaction on cooling effectiveness in the configuration considered.

Since film cooling involves heat transfer through the solid–fluid interface, it is inherently a conjugate heat transfer problem. A two-dimensional numerical simulation with multiphase modeling carried out by Bills et al. [29] showed that the accuracy of the conjugate heat transfer predictions of film cooling could be improved by incorporating the embedded coolant channel geometry and species composition. A comprehensive review of film cooling literature can be found in Shine and Shrinidhi [30].

This present study sets off with this background. A vast majority of the existing studies on film cooling pertain to subsonic or incompressible flow conditions on flat plates [23]. Studies on flat plate configurations—which dominate the literature on film cooling research—do not reflect the impact of the favorable pressure gradient which characterizes the accelerating flow field of a supersonic nozzle. The formation and growth of the mixing layer in the presence of the favorable pressure gradient (as in the nozzle) and pertinent aspects of the impact of the mixing zone on cooling in nozzles have not been discussed with an adequate focus in most of the previous studies. Additionally, the majority of the studies have been reported for temperatures less than 1200 K [31]. Nozzles in rocket engines are typically exposed to temperature levels in the range of 2500–3500 K. This high-temperature level constrains experimental investigations. While there are studies that address the optimization of injector configurations for better cooling effectiveness (e.g., [9,10]), they primarily focus on cylindrical injectors or elliptical injectors.

This present study is focused on the application of film cooling in rocket nozzles where the flow field is markedly influenced by (i) compressibility, (ii) the strongly favorable pressure gradient that exists along the nozzle, and (iii) the variable area of the cross-section. Each of these (which are coupled within themselves) can influence the progress of cooling along the nozzle wall. With an emphasis on exploring the means to improve cooling effectiveness, this study focuses on the development of the mixing layer and its impact on the temperature field. Conjugate heat transfer modeling [29] is used to comprehensively analyze the fluid-to-solid transfer of thermal energy. In line with the objectives, this study:

1. Explores the potential of a novel method of tangential injection through a diverging injector.
2. Analyses the growth of the mixing layer within the flow field of a supersonic nozzle—particularly, the growth of the thermal boundary layer is examined when the hot stream issues at a temperature of 2500 K.
3. Examines the impact of coolant species on mixing and cooling effectiveness by comparison of three coolant gases: three coolants (over a range of molecular masses) are considered to evaluate and compare their cooling performance in a supersonic nozzle, where the flow velocity varies from near-stagnation conditions to a Mach number of 3.0.

The diffuser-type injector configuration explored in this study for rocket nozzle film cooling is an improvisation over the reported methods of coolant injection.

An FVM-based commercially available CFD tool, ANSYS Fluent, is used for the simulations. The numerical model is first validated with published experimental data and then extended to cover the range of conditions and species identified for the scope of the analysis. This study covers a range of blowing ratios of practical interest for the three coolant fluids considered, Air, Neon and Helium. One of the major factors considered in this present study is film cooling performance in a compressible flow, as many of the relevant applications are in compressible flows. The three gases (Helium, Neon and Air) are selected to represent the respective ranges of molecular mass (4, 20 and 29, respectively) because molecular mass influences the expansion behavior in the nozzle as it directly influences the specific gas constant. The inclusion of Neon in this study was particularly in view of covering a range of molecular masses. The hot stream temperature is 2500 K.

## 2. Computational Methodology

The problem under consideration involves steady compressible flow through a convergent-divergent nozzle with multiple species. The density-based solver in Fluent was used to solve energy, momentum and continuity equations. The shear stress transport (SST) model with k-ω formulation was used for turbulence as its near-wall modeling approach makes it usable within the viscous sublayer as well. The SST formulation adopts a k-ε methodology in the free stream, thereby addressing the sensitivity of the standard k-ω model to the inlet free-stream turbulence properties [32]. Species transport without chemical reactions was used to model the mixing of the hot stream and the film coolant. The mathematical model consists of the conservation equations for each component species. The species mass fraction for each component was locally calculated by solving the respective convection–diffusion equation. The specific heat, viscosity and thermal conductivity were weighted by the mass of species to obtain mixture properties. Density variation was accounted for by the ideal gas equation. The implicit formulation in FLUENT was used to deal with the coupling between the flow field and the pressure field. Second-order upwind schemes were used for solving the governing equations. The use of the solver for modeling supersonic flows with diverse applications has been reported in several previous studies, including [33] and [34].

## 3. The Computational Domain and Boundary Conditions

**Figure 1:**
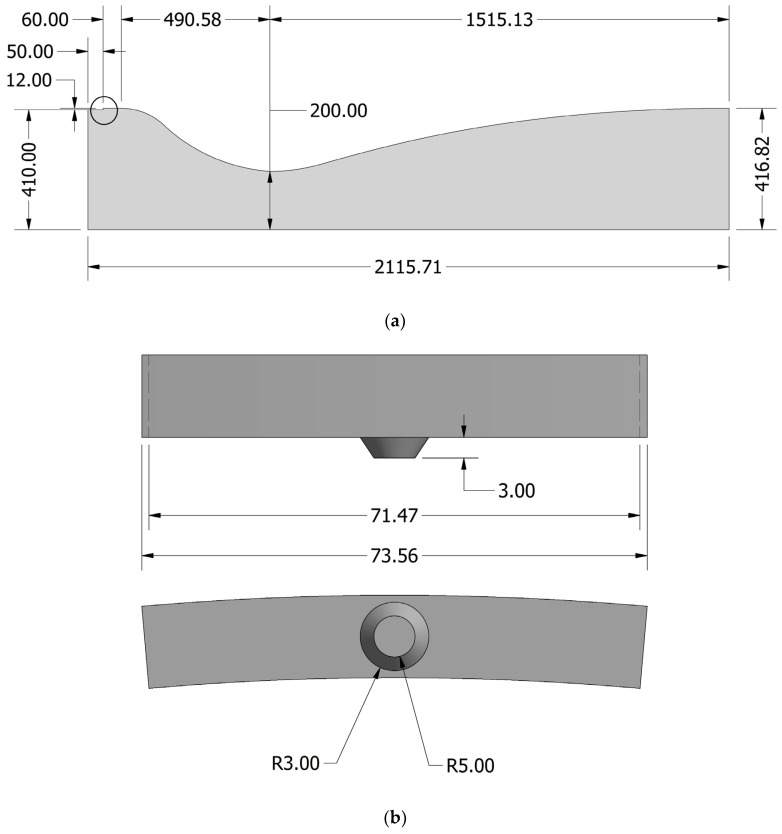
(**a**) Rocket nozzle dimensions. (**b**) Coolant injector for 30° taper angle dimensions (all dimensions are in mm).

A 3D bell-type nozzle geometry was designed using the Method of Characteristics (MOC) [35] based on the reference of the RS-68 Rocketdyne engine. The design was made for an exit Mach number of 3. A sector of the nozzle was deemed sufficient for analysis, as the nozzle is symmetric about the axis. This present study uses a 10° sector of the nozzle. The coolant injector is located 0.05 m downstream from the nozzle inlet. The coolant is injected tangentially along the wall using a diffuser-type injector. The schematic of the nozzle and the injector configuration for a taper angle of 30° is shown in Figure 1.

**Figure 2:**
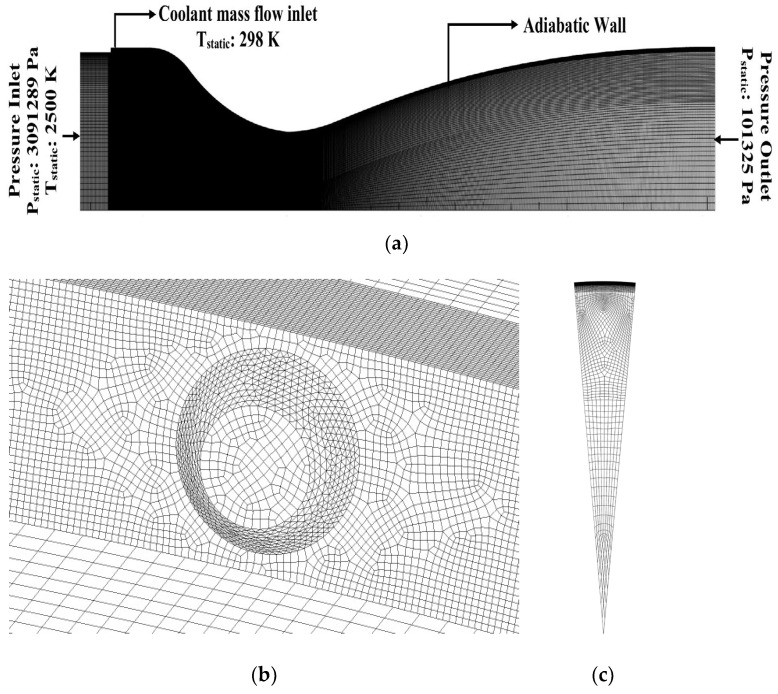
(**a**) Mesh along the symmetry plane and boundary conditions. (**b**) Mesh near the coolant injector. (**c**) Mesh on the nozzle inlet wedge section.

**Figure 3:**
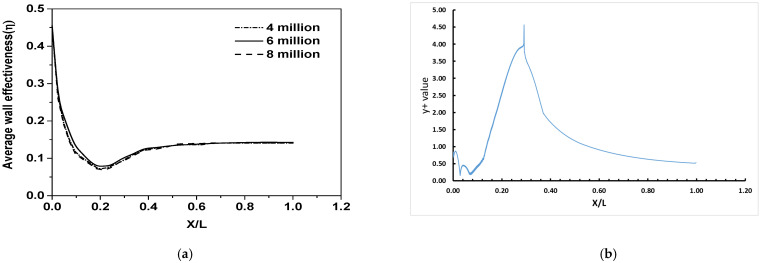
Grid independence study and y+ values along the line on the nozzle wall. (**a**) Grid independence of supersonic nozzle under consideration. (**b**) y+ value along a line on nozzle wall.

The domain is split into three regions: (1) the nozzle region before coolant injection, (2) the coolant injection zone in the convergent region extending to the throat, and (3) the expansion region after the throat of the nozzle. The grid points were closely clustered in the near wall region to adequately resolve the thin boundary layer. The entire fluid domain was discretized using hex-wedge elements. The nozzle symmetry mesh and the boundary zones (along with the conditions specified at the boundaries) are shown in Figure 2a. The mesh near the coolant injector region is shown in Figure 2b. A total of 1355 grid points were used along the length of the nozzle, whereas 50% of the grid points are clustered in the convergent section of the nozzle downstream of the injection location in order to resolve the mixing region adequately. The wedge section of the nozzle consists of 120 grid points radially and 40 grid points on the 10° arc of the wedge (Figure 2c). The first cell distance from the wall was fixed at 3.5 × 10^−6^ m, which leads to a maximum wall plus y^+^ of 5.07. Sample y+ values along the line on the supersonic nozzle wall under consideration in this present work are shown in Figure 3b.

The nozzle inlet gas is air at a temperature (*T_g_*) of 2500 K, and the coolant injection temperature (*T_c_*) is 298 K. The inlet stagnation pressure (*P*_0_) and the inlet static pressure (*P_s_*) were set to be 3.13 MPa and 3.09 MPa, respectively. The inlet static pressure (*P_cs_*) of the coolant injection was 3.09 MPa. This study investigates film cooling performance for four blowing ratios (BR): (i) 0.5, (ii) 1, (iii) 1.5 and (iv) 2, and for three different coolants: (i) Air, (ii) Neon and (iii) Helium.

## 4. Results and Discussion

### 4.1. Grid Independence Study and Validation

A grid refinement analysis for the geometry was carried out (boundary conditions are the same as those described below for the validation), and the results are shown in Figure 3a. For the 10° sector of the nozzle, three meshes with the following sizes were tested: (i) four million elements, (ii) six million elements and (iii) eight million elements. The mesh with six million elements was found to be adequate for a grid-independent solution and was used for further simulations.

For validating the numerical model, results from the experimental study of Lieu [36] were used. In their experiments, internal film cooling and regenerative cooling along the walls in a convergent-divergent nozzle were analyzed for a nozzle with an area ratio of 2.4:1 and a designed Mach number of 2.4. The boundary conditions in the validation study were specified exactly as in the experiments of [36]. At the inlet (of the hot stream), pressure boundary conditions were specified, corresponding to a stagnation pressure of 21.7 bar, along with a stagnation temperature of 673 K. Conductive heat transfer through the walls was modeled using the conjugate heat transfer approach [35]. The coolant air was injected at an angle of 10° with the mainstream with an inlet velocity of 136 m/s and at a temperature of 300 K. The regenerative coolant used was water (as in the experimental study [36]), which was made to flow through a channel parallel to the nozzle wall with an inlet stagnation pressure of 1 bar and stagnation temperature of 300 K.

**Figure 4:**
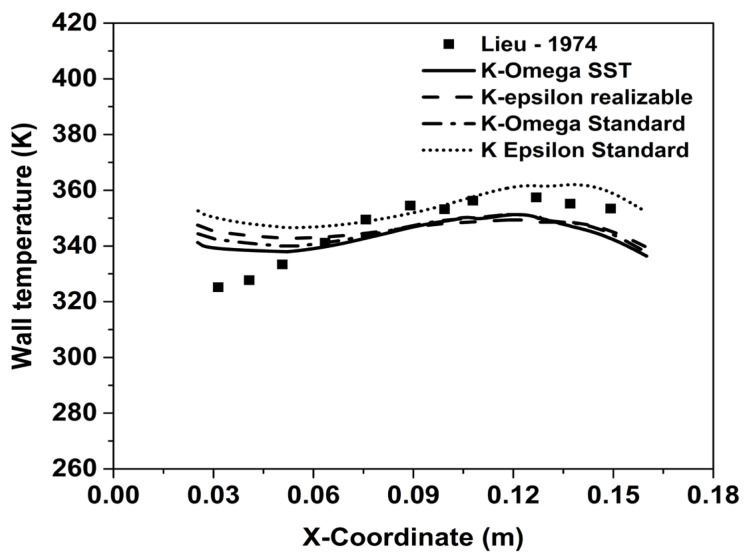
Comparison of simulated temperature distribution along the nozzle wall with experimental data from [26].

Figure 4 shows the wall temperature for the experimental study by Lieu [36] compared against the temperature distribution from the numerical simulations for different two-equation turbulence models. The k-ω model with SST (shear stress transport) formulation was found to provide better agreement with the experimental data. With the SST K-ω model, the deviation from the experimental values of [36] was within acceptable limits considering the experimental uncertainty. Quantitatively, the highest deviation from the experimental data was about 4%. This match is considered to be adequate for the purpose of this present study, and all the subsequent simulations reported here are carried out using the SST k-ω model. The choice of the SST k-ω model over the k-ε model was made primarily based on the comparison in the upstream location (closer to the point of injection).

### 4.2. Film Cooling Effectiveness

The film cooling effectiveness calculated along the nozzle wall is used to quantify the cooling performance. In line with the objective of this study, the film cooling effectiveness for Helium, Neon and Air was evaluated for different blowing ratios. The impact of the taper angle of the injector (as illustrated in Figure 1) is also examined. The *laterally averaged* values of adiabatic effectiveness were used for comparison of the cooling performance. The lateral averaging was made circumferentially along the 10° sector of the nozzle wall. The following definition, popularly used in film cooling literature, is made use of to calculate adiabatic cooling effectiveness:

$$\eta =\frac{T_{h}-T_{w}}{T_{h}-T_{c}}$$

In the above definition, *T_h_* indicates hot stream temperature, *T_w_* is the wall temperature of the cooled nozzle, and *T_c_* is the inlet temperature of the coolant.

#### 4.2.1. Effect of Injector Taper Angle on Cooling Effectiveness

**Figure 5:**
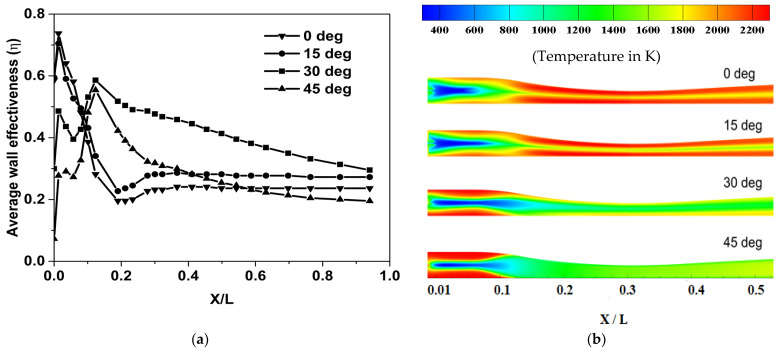
(**a**) Effect of injector taper angle on film cooling effectiveness. (**b**) Wall temperature contours of Helium coolant for different injector taper angles.

The effect of the taper angle of the injector on the film cooling effectiveness was studied for the following values (of taper angle): (i) 0°, (ii) 15°, (iii) 30° and (iv) 45°. The laterally averaged wall effectiveness is plotted (along the axial direction) for the four taper angles in Figure 5a, and the corresponding temperature distributions along the nozzle wall are shown in Figure 5b.

It can be seen from Figure 5b that an injector taper angle of 30° results in the most effective cooling among the configurations considered. The plots in Figure 5a show that the average wall effectiveness is high in the subsonic region of the nozzle (x/L < 0.3). Downstream of the throat, a drop in effectiveness is observed for all the injector configurations. It can be observed from Figure 5a that the taper angle of 0° has the steepest decrease in effectiveness near the throat. The temperature contours of 0° and 15° are qualitatively similar, as shown in Figure 5b, and the average film cooling effectiveness is also comparable for them (see Figure 5a). The temperature contours indicate that the low effectiveness for a taper angle of 0° can be attributed primarily to the lesser lateral spread of the coolant downstream of the location of injection. It can be inferred from the temperature contours that 45° has the highest lateral spread of coolant among all the injector taper angle configurations, and this excess spread of coolant also leads to a drop in the average film cooling effectiveness.

The above observations can be explained on the basis of two competing factors that influence the average cooling effectiveness: (1) the mixing of the coolant with the hot mainstream, which impedes the cooling at the wall, and (2) the lateral spread of the coolant jet is expected to improve the cooling. With a taper angle of 45°, the momentum of the coolant jet in the axial direction will be relatively lower (due to a higher lateral spread imposed by the injector geometry). This possibly enhances the rate of mixing in the transverse direction with the hot stream, which in turn reduces the extent of cooling at the wall. However, too low a taper angle (such as 0° and 15°) will also adversely impact the cooling because of the lower lateral spread, which reduces the circumferential area covered by the coolant jet. This points out the existence of an optimum taper angle for the injector, and for the values considered in this present study, 30° looks to be the most effective angle. In view of this, the remaining studies reported in this paper are carried out using the injector configuration with a taper angle of 30°.

#### 4.2.2. Effect of Coolant Properties and Blowing Ratio on Cooling Effectiveness

**Figure 6:**
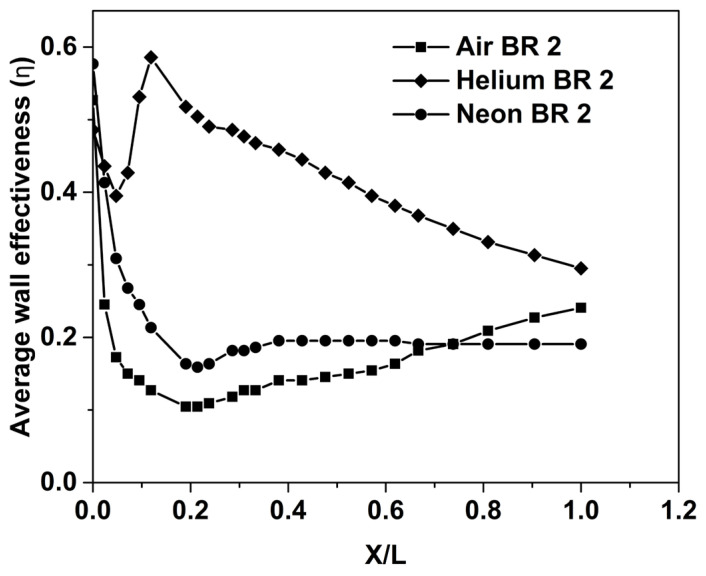
Cooling effectiveness for BR = 2.0.

The properties of coolant, such as density, thermal conductivity, specific heat and molecular mass, can have a significant impact on the cooling effectiveness [6]. In this present study, simulations are carried out in order to evaluate the impact of coolant properties on certain key aspects that govern the performance of a film cooling system as applied to a supersonic nozzle. In Figure 6, the variation of laterally averaged film cooling effectiveness along the nozzle wall is plotted for the three coolants considered, namely, Air, Helium and Neon, all for a blowing ratio of 2. It is clearly observed that Helium provides considerably better cooling compared to Air and Neon. Experiments by Parthasarathy et al. [37] on an axisymmetric center-body at a lower temperature level (around 450 K) have indicated Helium as having superior cooling performance over Air.

**Figure 7:**
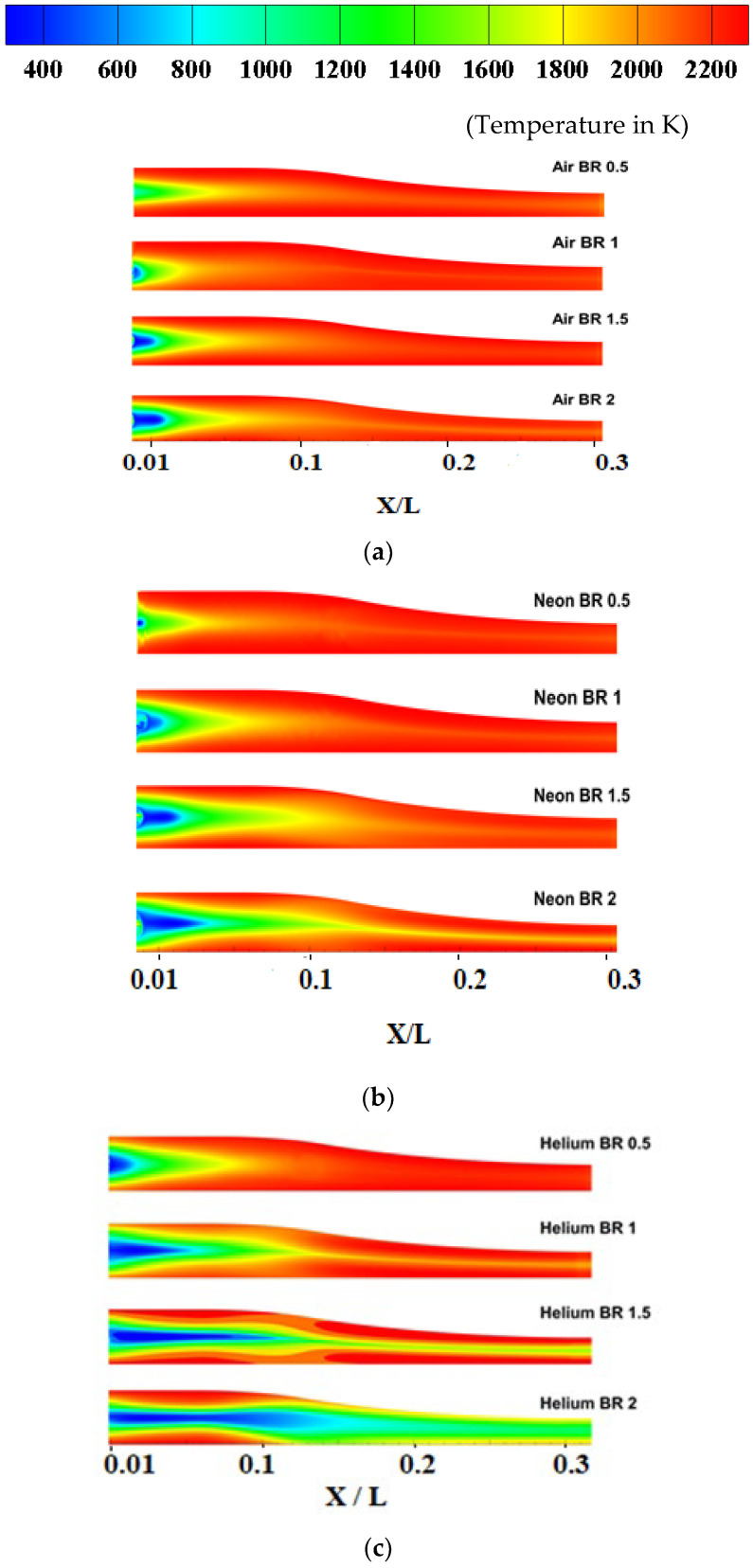
Comparison of wall temperatures for coolants: (**a**) Air, (**b**) Neon, (**c**) Helium.

While Helium has higher thermal conductivity than Air, its specific heat (at constant pressure) is about five times that of Air; hence, the resulting increase in the temperature of the Helium layer will be relatively lower. This provides a better cooling performance. Furthermore, the variation of the effectiveness of Helium in the stream-wise direction can be corroborated with the spreading rate of the jet as reflected by the temperature distribution shown in Figure 7c. Peak effectiveness of Helium is observed at X/L = 0.2. From Figure 7c, it can be seen that at X/L = 0.2, the lateral spreading of the Helium jet increases, and there is a corresponding increase in effectiveness. As stated above, the thermophysical properties of Helium are favorable to the cooling enhancement, and this increased rate can be attributed as the reason for the difference in the qualitative variation of effectiveness for Helium (at this blowing ratio). There is a significant drop in the effectiveness of Helium after X/L = 0.2 (Figure 6)—this can probably be attributed to excessive spreading, which leads to a concomitant increase in transverse mixing as well.

Similar variations in the axial distribution of effectiveness can be observed for the other two coolants as well (Figure 6). The locations of the reversal of trend vary because of the difference in gas properties and the impact of compressibility. The cooling effectiveness for Air starts to increase after X/L = 0.3, while the effectiveness of the other two coolants decreases around the same location. This difference may be attributed to the fact that in the case of He and Ne, the coolant is different from the mainstream gas (Air), and the rate of expansion will be different as well. When Air is used as the coolant, the hot gas and the coolant undergo expansion at the same rate.

**Figure 8:**
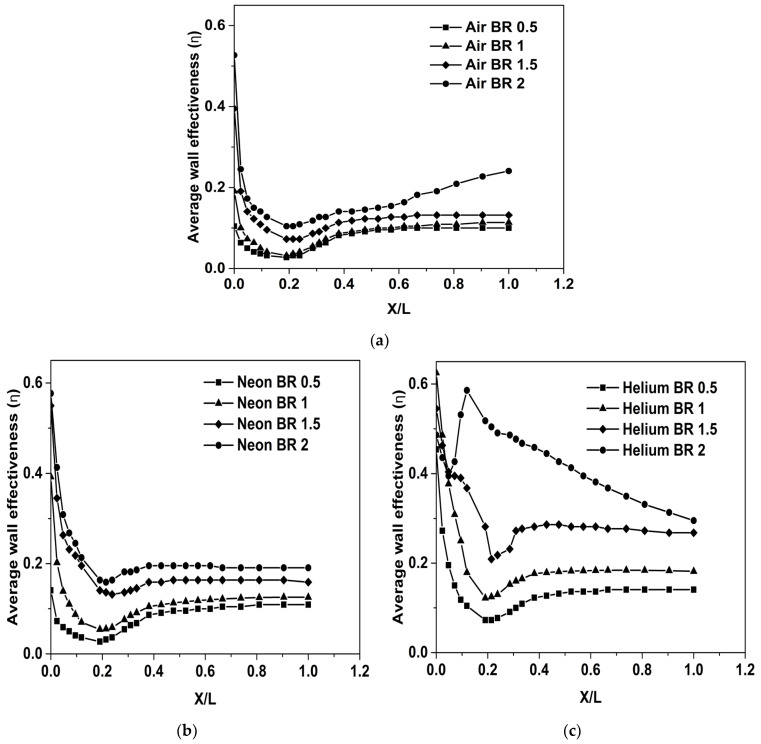
Cooling effectiveness for: (**a**) Air, (**b**) Neon, (**c**) Helium.

The variation of the film cooling performance of Air, Neon and Helium for different blowing ratios is shown in Figure 8a–c, respectively. It is observed that film cooling effectiveness increases with an increase in blowing ratio for all three coolants. Additionally, the variation in film cooling effectiveness with respect to the blowing ratio is more predominant for Helium. This is because Helium is the lightest gas of the three (lowest density), and, as a result, the increase in velocity for a given increase in BR will be the highest for Helium. For relatively low-velocity applications such as turbine blade cooling, the impact of the blowing ratio on cooling has been analyzed by previous studies [38].

### 4.3. Mixing Layer Analysis

The interaction or the mixing of coolant and mainstream occurs in a small region near the interface of both gases, defined as the *mixing layer*. There are different methods (using velocity, temperature, mass fraction) used to characterize and analyze the mixing layer. Here we first consider thermal mixing using temperature distribution within the pertinent zone to quantify the extent of the mixing layer. The mixing layer thickness influences the rate of heat transfer between the mainstream and the coolant gases, which in turn impacts the variation of film cooling effectiveness along the length of the nozzle. Specific locations along the axial direction are identified to characterize the development of the mixing layer. The mixing layer was characterized using the parameter τ defined as follows:

$$\tau =\frac{T_{L}-T_{Lmin}}{T_{Lmax}-T_{Lmin}}$$

**Figure 9:**
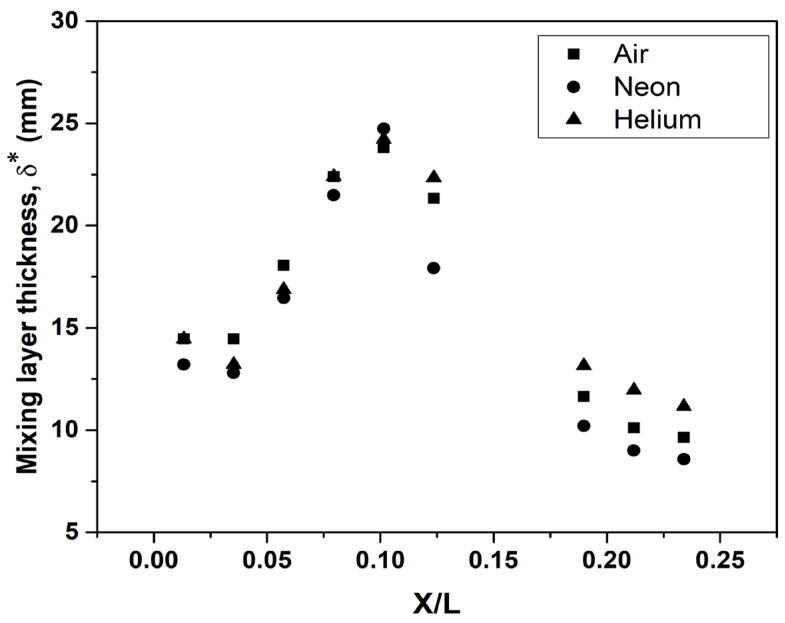
Mixing layer thickness for blowing ratio of 2.

The subscripts *L_min_* and *L_max_* for temperature (*T*) in the above definition indicate the local minimum and maximum values of temperature, respectively, at the specified station. This definition enables us to ascribe the point of τ=0 to the location of coolant temperature and τ=1 to the point where the temperature is equal to the mainstream value (hot gas temperature). The thickness of the mixing layer (δ*) is identified as the transverse location (as measured from the wall) where the value of τ is equal to unity (it may be noted that since temperature varies along the nozzle, τ has to be evaluated at each axial location). Values of the mixing layer thickness for different coolants are shown in Figure 9. The thickness is less near the coolant injector region due to low initial mixing between the mainstream and the coolant. The mixing layer thickness then increases as the entrainment of the mainstream into the coolant increases. The thickness peaks around X/L = 0.2 and then steadily decreases as the flow approaches the throat. It can be seen that, after X/L = 0.2, the mixing layer thickness of Helium is higher than the other two coolants. This thick mixing layer is also responsible for the higher film cooling effectiveness of Helium.

#### 4.3.1. Temperature Profiles Inside the Mixing Layer

**Figure 10:**
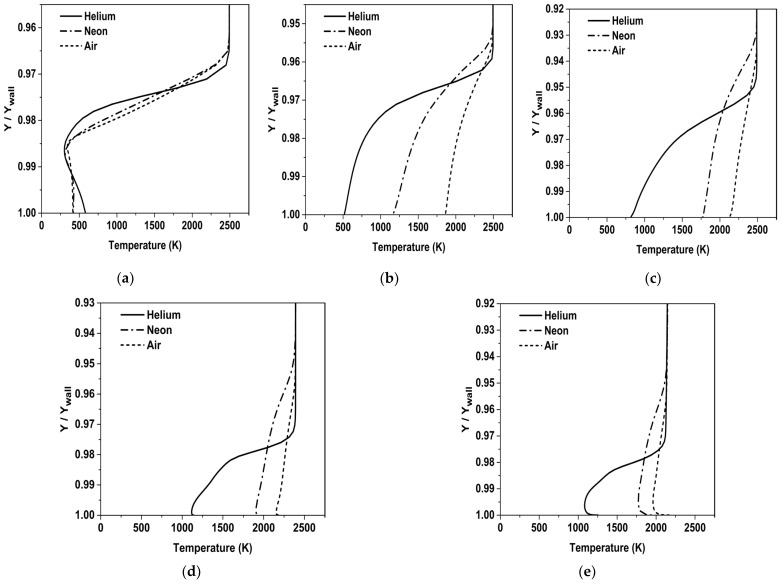
Variation of temperature inside mixing layer at different stations. (**a**) X/L = 0.01, (**b**) X/L = 0.05, (**c**) X/L = 0.12, (**d**) X/L = 0.21, (**e**) X/L = 0.3.

The temperature profiles are analyzed at the following axial stations: X/L = 0.01, 0.05, 0.12, 0.21 and 0.3. The Y coordinate is normalized by the height of the wall at that axial station, as measured from the axis. The variation of the temperature inside the mixing layer is shown in Figure 10. This indicates that the mixing layer temperature is the lowest for the Helium coolant at all the stations. The lower mixing layer temperature of Helium stems from its physical properties, such as high specific heat capacity and low molecular mass. Progressive mixing manifests as a sudden steep change of slope in the temperature profile of Helium. This change in slope is not so prominent for Neon and Air. The temperature of the coolant gradually increases as the coolant approaches the throat, mainly due to mixing with the mainstream.

#### 4.3.2. Velocity Profiles Inside the Mixing Layer

**Figure 11:**
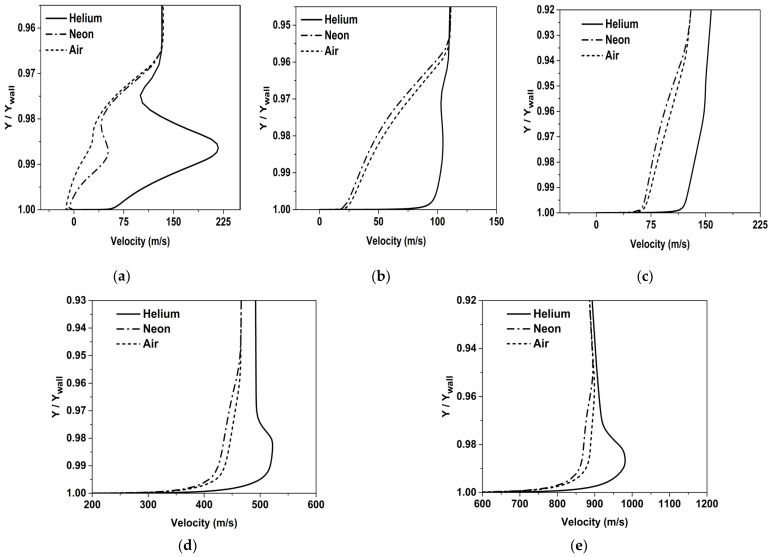
Variation of velocity inside mixing layer at different stations. (**a**) X/L = 0.01, (b) X/L = 0.05, (**c**) X/L = 0.12, (**d**) X/L = 0.21, (**e**) X/L = 0.3.

In Figure 11, the variation of velocity in the mixing layer is shown. It can be observed that the initial velocity of Helium is higher than that of the other two coolants. Owing to its lower density, Helium has to be injected at a higher velocity in order to maintain the blowing ratio. It can also be seen that the velocity of Helium at stations X/L = 0.05 and X/L = 0.12 is almost comparable to the velocity of the mainstream. The Helium coolant initially has a high velocity, and downstream to the injection, the velocity eventually equalizes to the mainstream velocity due to momentum transfer. In the converging section, Helium accelerates at a higher rate compared to the mainstream due to its low density. Despite the subsequent increase in momentum mixing, Helium still maintains its superior cooling performance due to its thermophysical properties, as discussed earlier.

#### 4.3.3. Mass Fraction Profiles Inside the Mixing Layer

**Figure 12:**
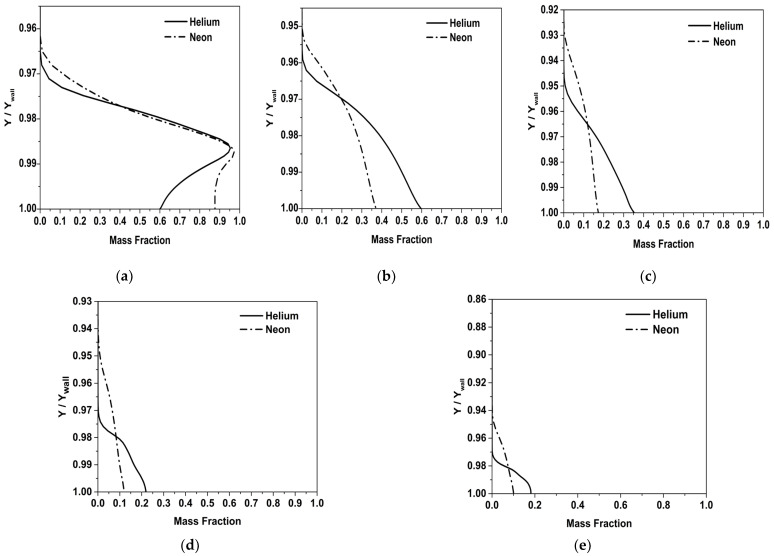
Variation of mass fraction inside mixing layer at different stations. (**a**) X/L = 0.01, (**b**) X/L = 0.05, (**c**) X/L = 0.12, (**d**) X/L = 0.21, (**e**) X/L = 0.3.

In Figure 12, the variation of mass fraction in the mixing layer at different locations along the nozzle axis is shown. It can be seen that the mass fractions of Helium and Neon decrease gradually as the flow approaches the throat. The decrease in the mass fraction is concomitant to the increase in mixing between the mainstream and the coolant. The variations along the axial direction are indicative of species diffusion resulting in the coolant mixing with the hot stream, which in turn adversely impacts the cooling performance. It can be seen that the mass fraction of Neon decreases at a faster rate compared to Helium. This suggests that the rate of species mixing (mixing at the molecular level) between Neon and the mainstream is higher than that for Helium. A comparatively high fraction of Helium is maintained while the flow reaches the throat, and hence, the effectiveness of Helium is higher compared to Neon and Air.

## 5. Conclusions

Film cooling in a supersonic nozzle was studied under thermal and flow conditions comparable to those of a rocket nozzle, with an emphasis on the improvement of cooling effectiveness. Species modeling was incorporated to analyze the cooling performance of three different coolants: (i) Air, (ii) Neon and (iii) Helium over a range of blowing ratios. Parameters influencing the effectiveness, such as coolant injector configuration (for coolant injector taper angles: 0°, 15°, 30° and 45°) and mixing layer, were analyzed. The following are the major observations made in this study:

1. In comparison to the conventional cylindrical injectors (0°), diffuser-shaped injectors were found to provide a more lateral spread of the coolant and increase the wall effectiveness. There are indications of the possibility of an optimum angle of injection, which provides sufficient lateral spreading without compromising the effectiveness. The 30° taper angle coolant injector was found to be an optimal configuration in this present study.
2. The mixing layer thickness was determined at nine different stations in the subsonic region of the nozzle. Downstream of the coolant injection, the diffusion of the hot stream into the coolant flow can be visualized by the progressive decline in the mixing layer thickness. The thick mixing layer of Helium is contributive to its higher film cooling effectiveness, as the convection of heat from the mainstream to the Helium coolant will be at a lower rate.
3. The mass fraction profiles indicate that the mass fraction of Neon near the wall decreases at a faster rate compared to Helium. This suggests that the rate of entrainment of the mainstream into Neon is higher than the rate of entrainment for Helium.

## Data Availability

Not applicable.
